# Proteomic study of sporadic inclusion body myositis

**DOI:** 10.1186/s12953-014-0045-2

**Published:** 2014-09-12

**Authors:** Ke Li, Chuanqiang Pu, Xusheng Huang, Jiexiao Liu, Yanling Mao, Xianghui Lu

**Affiliations:** Department of Geriatric Neurology, Chinese PLA General Hospital, Beijing, 100853 China; Department of Neurology, Chinese PLA General Hospital, 28 Fuxing Road, Beijing, 100853 China

**Keywords:** APP, αB-crystallin, Neurogenic muscular atrophy, Sporadic inclusion body myositis

## Abstract

**Background:**

Sporadic inclusion body myositis (s-IBM) is the most commonly occurring acquired inflammatory myopathy in elderly people (>45 years); however, pathogenic mechanisms are poorly understood and diagnostic tools are limited. In view of this, new therapeutic and diagnostic molecular markers for s-IBM need to be identified.

**Experimental design:**

In this study, the proteomes from three s-IBM cases were compared with those from three cases of neurogenic muscular atrophy (control). Proteins were separated by 2-dimensional polyacrylamide gel electrophoresis and profiled by mass spectrometric sequencing and subsequently validated by western blot.

**Results:**

Differential expression was noted in 29 proteins (16 upregulated and 13 downregulated) in s-IBM compared with the control group. Functions of these proteins include oxidative stress response, regulation of apoptosis, signal transduction, and cytoskeleton. Expression of both amyloid precursor protein (APP) and αB-crystallin was increased in s-IBM cases.

**Conclusions:**

Our study reveals a unique pattern of protein expression in s-IBM, which should be further investigated in a wider cohort of IBM patients to fully realize the potential diagnostic or therapeutic benefits.

## Background

Sporadic inclusion body myositis (s-IBM) is a sporadic inflammatory muscle disease that occurs most frequently in middle-aged and elderly individuals [1]. There is considerable debate as to whether the pathogenesis of s-IBM is a T cell mediated autoimmune myopathy, or a myodegenerative disorder of undetermined type with a secondary inflammatory component [2]. Moreover, an effective treatment for s-IBM does not exist; thus, current research efforts are focused on identifying molecular markers for this disease.

Until now no proteomic studies on s-IBM muscular tissue have been carried out in China. Here, we investigated the proteome of s-IBM muscular tissue with the goal of finding specific protein markers closely associated with development and morbidity of s-IBM, which in turn may provide new clues for the pathogenesis of the disease.

## Results and discussion

**Figure 1:**
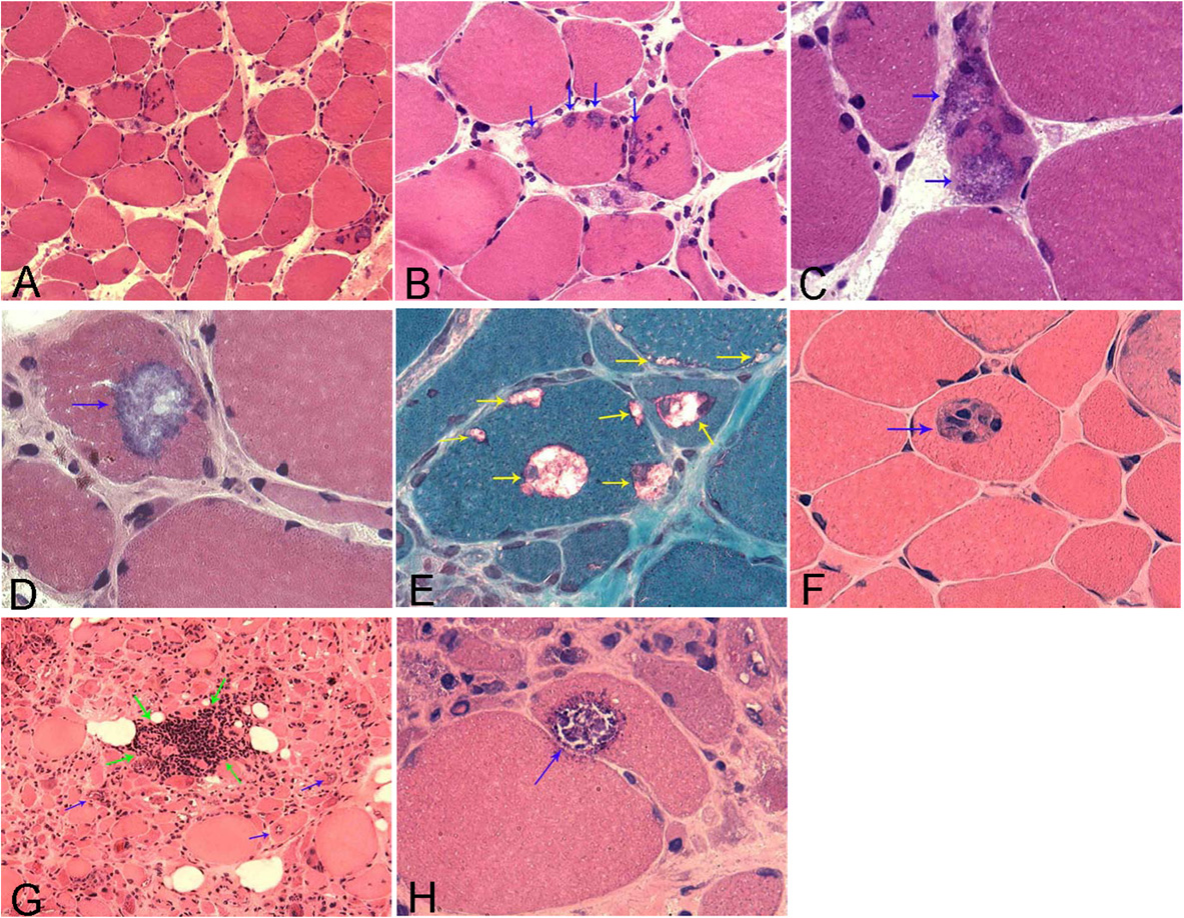
**Pathological characteristics of sporadic inclusion body myositis (s-IBM). A-C**: Case 1, atrophic muscle fibers were angular, irregular, and round, with muscle fiber hypertrophy, HE staining, ×100 **(A)**; Fimbriated cavity and inclusion bodies (blue arrows), HE staining, ×200 **(B)**; Sand-like particles (inclusion bodies) (blue arrows), HE staining, ×400 **(C)**. **D** ~ **F**: Case 2, round Fimbriated cavities in the atrophic muscle fibers, with sand-like inclusion bodies in which (blue arrows), HE staining, ×400 **(D)**; Red particles of inclusion bodies in fimbriated cavities (yellow arrows), Gomori staining, ×400 **(E)**; Nonnecrotic muscle fibers infiltrated by monocytes (blue arrows), HE staining, ×200 **(F)**. **G **~ **H**: Case 3, Focal inflammatory infiltration (green arrows) and fimbriated cavities showed in several muscle fibers (blue arrows), HE staining, ×100 **(G)**; Round fimbriated cavities in atrophic muscle fibers, with gross particles of inclusion bodies in which (blue arrows), HE staining, ×400 **(H)**.

Clinical characteristics of the cases with s-IBM are summarized in Table 1. The involved muscles included quadriceps femoris, iliopsoas, finger flexor muscles, biceps brachii, tibialis anterior, and deltoid. CK levels in the serum of the patients were 384 IU/L, 234 IU/L, and 445 IU/L, respectively. Biopsy sites of the patients were biceps brachii or quadriceps femoris, whereas biceps brachii was used for the normal tissue specimens. Pathological examination showed that atrophic muscle fibers were angular, irregular, and round, with muscle fiber hypertrophy. Fimbriated cavity and sand-like particles inclusion bodies were shown in the atrophic muscle fibers. Non-necrotic muscle fibers were infiltrated by monocytes (Figure 1).

**Table 1:** **Clinical characteristics of the cases with s-IBM**

**Cases**	**Sex**	**Age at onset**	**Duration(year)**	**Symptoms at onset**	**Involved muscles**	**CK (IU/L)**	**EMG**	**Biopsy site**
1	Male	52	2.5	Weakness of both upper limbs	Qua, Bi, Fin, Del	384	Myogenic	Bi
2	Female	60	1.2	Weakness of both lower limbs	Qua, Bi, Fin	234	Myogenic	Qua
3	Male	51	3	Weakness of right lower limb	Qua, Bi, Fin, Ilio, TA	445	Myogenic	Bi

Qua: quadriceps femoris; Ilio: iliopsoas; Fin: finger flexor muscles; Bi: biceps brachii; TA: tibialis anterior; Del: deltoid.

**Figure 2:**
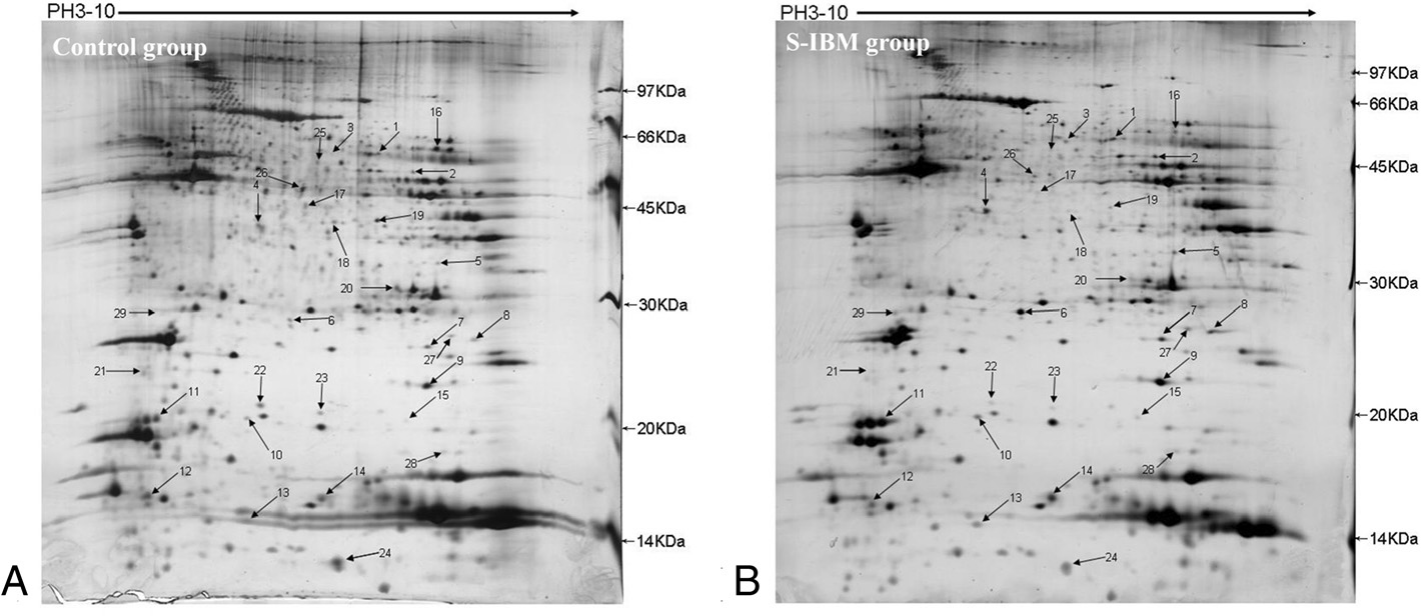
**Two-dimensional electrophoresis of muscle tissue from control group (A) and s-IBM patients (B).** Arrows represent the protein spots with significant differences in relative intensity, identified by t-test. The protein spots with significant differences in expression are numbered #1-29.

IPG dry adhesive tape (18 cm, pH 3–10) was used in the first dimension of electrophoresis, while gel porosity was 13% and the molecular weight gradient was 14–97 kDa in the second phase (Figure 2). These parameters resulted in clear protein separation, and uniformity of protein distribution. Each sample was subjected to electrophoresis twice, with protein spots in the duplicate maps demonstrating good reproducibility and stability. The average matching rate was as high as 85.1%, and the number of detected matching protein spots was 1052. Using ImageMaster 2D Platinum software, 29 differential protein spots were determined to have significant changes compared with controls (*P* < 0.05). Of these, 16 proteins showed increased expression in s-IBM, while the remaining 13 showed decreased expression.

**Figure 3:**
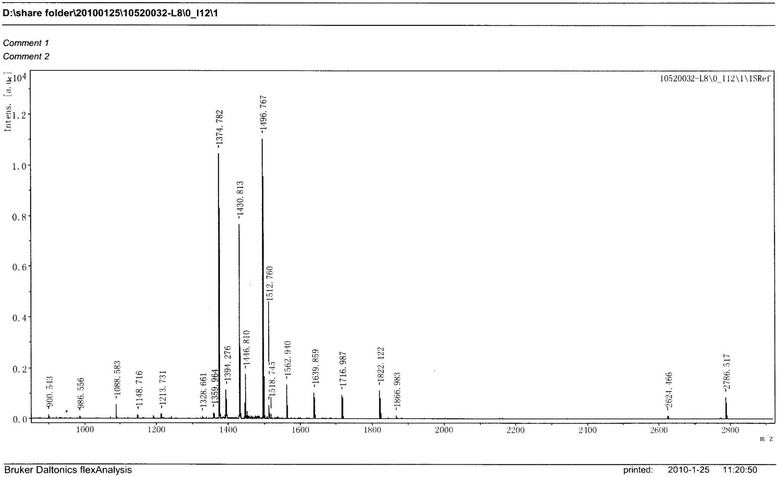
**Representative electrophoretogram of peptide mass of Spot 9.**

The 29 proteins with differential expression were chosen from the s-IBM group and subjected to MALDI-TOF-MS analysis post trypsin-digestion. A representative electrophoretogram of peptide mass from Spot 9 is presented in Figure 3. Proteins were identified using the Mascot database (Table 2).

**Table 2:** **Search results of proteins with differential expression using Mascot database**

**Spot No.**	**Protein name**	Score*	**Chromosome**	**NCBI index**	**Expression**	**Function**
1	Aldehyde dehydrogenase 1 family, member A1	141	9q21.13	gi|21361176	↑	Oxidation reduction
2	Enolase 1, (alpha)	218	1p36.3-p36.2	gi|4503571	↑	Negative regulation of cell growth
3	Amyloid beta A4 protein precursor	80	21q21.2;21q21.3	gi|4502167	↑	Neuron apoptosis, ion binding
4	Troponin T type 1 (skeletal, slow)	165	19q13.4	gi|187173290	↑	Troponin T binding, muscle contraction
5	Voltage-dependent anion channel 2	130	10q22	gi|55664661	↑	Ion channel activity
6	Myosin, light chain 6B, alkali, smooth muscle and non-muscle	230	12q13.13	gi|4505303	↑	Muscle contraction
7	Superoxide dismutase 2, mitochondrial	127	6q25.3	gi|67782305	↑	Antioxidant activity
8	Peroxiredoxin 1	230	1p34.1	gi|4505591	↑	Oxidoreductase activity
9	Crystallin, alpha B	172	11q22.3-q23.1	gi|4503057	↑	Response to reactive oxygen species
10	Heat shock protein, alpha-crystallin-related, B6	70	19q13.12	gi|21389433	↑	Response to temperature stimulus
11	Myosin, light chain 2, regulatory, cardiac, slow	248	12q24.11	gi|94981553	↑	Regulation of muscle contraction
12	Cytochrome c oxidase subunit Va	61	15q24.1	gi|190885499	↓	Oxidation reduction
13	S100 calcium binding protein A13	76	1q21	gi|62632859	↑	Calcium ion binding
14	Fatty acid binding protein 3	147	1p33-p32	gi|4758328	↑	Lipid transporter activity
15	Transgelin	164	11q23.2	gi|48255905	↑	Actin binding
16	Fibrinogen beta chain	280	4q28	gi|70906435	↓	Cell activation, protein complex assembly
17	Myosin heavy chain IIa	125	17p13.1	gi|153791586	↓	Muscle contraction
18	Enolase 3 (beta, muscle)	72	17pter-p11	gi|119610782	↓	Phosphopyruvate hydratase activity, skeletal muscle regeneration
19	Troponin T3, skeletal, fast isoform 2	111	11p15.5	gi|112789538	↓	Muscle contraction
20	Carbonic anhydrase I	80	8q13-q22.1	gi|4502517	↓	Carbonate dehydratase activity, ion binding
21	Uridine monophosphate synthetase	68	3q13	gi|197210454	↓	Pyrimidine nucleoside metabolic process
22	Adaptor-related protein complex 3, mu 2 subunit	127	8p11.2	gi|5803000	↓	Protein transport
23	Haptoglobin-related protein	60	16q22.1	gi|296653	↓	Proteolysis
24	Hemoglobin beta	106	11p15.5	gi|75491671	↓	Oxygen transporter activity
25	Albumin	141	4q11-q13	gi|27692693	↓	Response to starvation, antioxidant activity
26	Myosin, heavy chain 7, cardiac muscle, beta	122	14q12	gi|386973	↓	Muscle contraction
27	Troponin I type 1 (skeletal, slow)	162	1q31.3	gi|14787427	↓	Regulation of muscle contraction
28	Chain A, Human peroxiredoxin 5	114	11q13	gi|6912238	↑	Oxidoreductase activity
29	Rho GDP dissociation inhibitor (GDI) alpha	83	17q25.3	gi|4757768	↑	Small GTPase regulator activity

*Mascot score (Mascot score > 65; *P* < 0.05, the P-value has been generated by the DAVID algorithm and were adjusted for multiple comparisons).

Entrez Gene IDs of the differentially expressed proteins were submitted on-line to the DAVID network station (National Institutes of Health (NIH)), and the biological functions of these proteins were classified using the Functional Annotation tool. The major biological functions included cytoskeleton organization, muscle contraction, regulation of muscle contraction, oxidative stress response, maintenance of homeostasis, regulation of apoptosis, and local adherence. Of these functional categories, 10 of the 29 proteins were involved in cytoskeleton organization, 7 participated in regulating apoptosis, and 5 proteins played a role in peroxidation (Table 3). We analyzed the KEGG signaling pathway, and found that the differentially expressed proteins were involved in a total of 19 signaling pathways (Table 4).

**Table 3:** **Major functions of proteins with differential expression**

**Function annotation**	**Gene number**	**Spot No.**	**P-value**
Biology process			
Muscle contraction	9	9, 17, 26, 6, 4, 19, 10, 27, 11	1.1E-5
Cytoskeleton	10	29, 3, 9, 17, 26, 11, 6, 4, 19, 27	3.9E-4
Regulation of apoptosis	7	29, 25, 3, 9, 8, 28, 7	4.4E-3
Negative regulation of apoptosis	5	29, 25, 9, 28, 7	5.0E-3
Response to reactive oxygen species	5	9, 26, 8, 28, 7	1.4E-5
Regulation of muscle contraction	5	11, 27, 4, 19, 10	3.9E-4
Cellular homeostasis	5	3, 23, 8, 28, 7	1.3E-2
Cytoskeleton-dependent intracellular transport	4	3, 17, 26, 6	1.5E-4
Stress-activated MAPK cascade	2	9, 8	2E-4
Molecular function			
Cytoskeletal protein binding	8	9, 17, 26, 11, 15, 27, 4, 19	4.4E-5
Pyrophosphatase activity	5	17, 11, 6, 26, 19	2.0E-2
Oxidoreductase	5	1, 12, 8, 28, 7	8.5E-3
Antioxidant activity	4	25, 8, 28, 7	1.1E-4
Glucose metabolic process	3	9, 2, 18	3.7E-2
Phosphopyruvate hydratase activity	2	18, 2	1.0E-2
Focal adhesion	2	17, 26	1.2E-2

Table 3 represent a list of KEGG associated to single protein changed in sIBM, related enriched protein are reported in Table 2, but are not consistent. For example, for proteins 11, 12, 26 associated to muscle contraction signal pathway, 11 and 26 are also reported in Table 2 (proteins associated to biological function of muscle contraction). For 11, 17, 26 associated to Tight junction signal pathway, 17 and 26 are also reported in Table 2 (proteins associated to biological function of focal adhesion).

**Table 4:** **Summary of proteins with differential expression participated in KEGG signal pathway**

**KEGG pathway**	**Gene number**	**Spot No.**
Muscle contraction	3	12, 26, 11
Tight junction	3	17, 26, 11
Huntington's disease	3	12, 7, 5
Parkinson's disease	3	12, 5
Alzheimer's disease	2	3, 12
Glycolysis/Gluconeogenesis, RNA degradation	2	2, 18
Neurotrophin signaling pathway	1	29
Lysosome	1	22
Retinol metabolism	1	1
Nitrogen metabolism	1	20
Oxidative phosphorylation	1	12
PPAR signaling pathway	1	14
Complement and coagulation cascades	1	16
Focal adhesion	1	11
Leukocyte transendothelial migration	1	11
Regulation of actin cytoskeleton	1	11
Vascular smooth muscle contraction	1	6
Pyrimidine metabolism	1	21
Calcium signaling pathway	1	5

**Figure 4:**
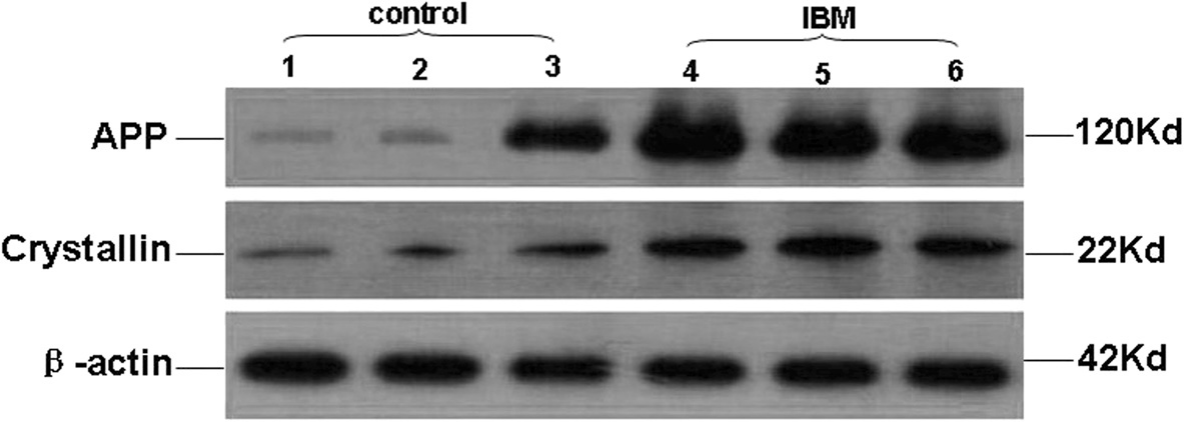
**Western blot of αB-crystallin and amyloid precursor protein expression in s-IBM.** Through semi-quantitative analysis using gray-scale quantification, steady-state expression levels of αB-crystallin and APP in s-IBM were found to be significantly increased relative to controls (P < 0.05). β-actin was used as an internal control, and protein expression was normalized by taking the target protein/β-actin.

By integrating bioinformatics analysis and literature search results, we selected APP and αB-crystallin from the differentially expressed proteins for further analysis via Western Blot. These results confirmed that expression levels of APP and αB-crystallin protein were increased in s-IBM tissue relative to normal muscle, validating our 2D proteome dataset. (Figure 4, Table 5).

**Table 5:** **Western blot results**

**Group**	**n**	**αB-crystallin/β-actin**	**APP/β-actin**
s-IBM	3	84.31 ± 8.29*	204.1 ± 12.43*
Normal control	3	22.27 ± 15.75	48.64 ± 69.67

*Compared with normal control group, *P* < 0.05.

Relative expression levels of αB-crystallin and APP mRNA in tissue from the s-IBM group were shown in Table 6. Similar to the results for protein levels, relative mRNA expression levels of both αB-crystallin and APP in the s-IBM group were significantly increased (P <0.05).

**Table 6:** **RT-PCR results**

**Group**	**n**	**αB-crystallin/β-actin**	**APP/β-actin**
sIBM	3	2.27 ± 0.81*	4.29 ± 1.15*
Normal control	3	0.92 ± 0.12	1.24 ± 0.63

*Compared with normal control group, *P* < 0.05.

The myodegenerative disease s-IBM is common in senile patients, and it is thought that ageing associated accumulation and sedimentation of proteins in intra-cellular environment plays a role in the disease pathogenesis [1,2]. Oxidative stress and stress reaction in endoplasmic reticulum also contribute to the disease pathogenesis. The above factors cumulatively lead to progressive degeneration and necrosis of muscle [1,2]. It was initially believed that s-IBM was a disease involving abnormal protein conformation, inclusive of unfolding and mis-folding of many proteins in muscle cells, which in turn induced cytotoxicity and abnormal accumulation of many proteins [3,4]. Therefore, investigation of proteins with differential expression during pathogenesis of s-IBM will provide the foundation for further revealing the pathological mechanisms of s-IBM. Proteomics can be used to screen a great quantity of differential protein spots in the target tissue, which will provide technical support for analysis of proteins with differential expression.

Hutchinson et al. (2008) did not find either distinctive expression or lack of protein expression between five cases of normal muscle tissues and six cases of IBM muscle tissues [5]. Of note though, the analyses was essentially based on present/absent criteria since the intensity of the protein spots were not analyzed in details, as in the current study, to detect differential expression of proteins, if any [5]. In the current study the electrophoretograms had good stability and reproducibility; in addition, the protein spots in the two electrophoretograms of the same sample showed good stability and reproducibility, and the average match rate was 85.1%. This allowed us to further analyze, based on the intensity of the spots, the differentially expressed proteins between IBM tissues and normal tissues. In a separate study, Li J et al. (2006) had compared the 2D electrophoresis results of four cases of IBM muscle tissues and five cases of muscle tissues of other myositis, and found that APP was one of the upregulated proteins in sIBM cases [6]. In comparison, we compared differential expression between IBM and normal muscle tissues and also found APP to be upregulated in sIBM cases. In addition, we also found αB-crystallin to be upregulated in these cases. APP is closely related to abnormal accumulation and mis-folding of proteins, whereas αB-crystallin is related to protein folding, hence suggesting a direct role of these proteins in the pathogenesis of sIBM.

Functional fuzzy grouping analysis was performed using the Functional Annotation tool supplied by the DAVID network. It was found that these proteins primarily participated in the cellular peroxidation response to stress, apoptosis regulation, signal transduction, and skeletal muscle contraction. Based on these major biological functions, the differentially expressed proteins can be classified as followed.

Proteins participating in oxidative stress responses include superoxide dismutase (SOD), peroxydase 1, αB-crystallin, the H-chain 7 of myoglobulin, the A- chain of peroxydase 5, and cytochrome C oxidase. αB-crystallin is a member of the small heat shock protein (HSP) family [7]. Additionally, it is expressed in many tissues, with particularly high levels found in cells with low rates of mitotic division, such as those found in crystalline lens, cardiac muscle, and skeletal muscle. It was reported that as a molecular chaperone protein, αB-crystallin mainly interacts with unfolded proteins, which inhibits coalescence and thus prevents accumulation of defective proteins [8]. It has also been found that in addition to functioning as a chaperone, αB-crystallin also played a role in inhibiting apoptosis under certain circumstances [9,10]. Previous work demonstrated that high expression of αB-crystallin was correlated with degenerative diseases, such as Alexander's disease [11], Alzheimer's disease (AD) [12], amyotrophic lateral sclerosis [13], Pick’s disease [14], Creutzfeldt-Jakob disease [15], and multiple sclerosis [16]. Our study showed increased expression of αB-crystallin in s-IBM, which indicated that this protein may be involved in the pathogenesis of s-IBM.

Proteins involved in regulation of apoptosis include amyloid precursor protein, SOD, peroxydase1, αB-crystallin, albumin, A-chain of peroxidase 5, and Rho GDP dissociation inhibiting factor-α. Amyloid precursor protein and peroxydase1 play a role in promoting apoptosis, while the others are involved in inhibiting apoptosis. Except for albumin, expression levels of the other four inhibitory proteins were increased. We hypothesize that while the organism activates anti-apoptosis mechanisms during progression of s-IBM, apoptotic activity cannot be fully suppressed, thereby leading to further development of the disease.

APP has been identified as the major protein in the plaques of senile AD patients [17], and mutation of this gene is considered an important etiological factor of AD. Our study showed by quantitative analysis that expression of APP protein, as well as mRNA, is increased in s-IBM patients, suggesting that APP participates in the pathophysiological progress of abnormal deposition into inclusion bodies. APP can induce endoplasmic reticulum stress (ERS), which subsequently induces the unfolded protein response (UPR). Schmidt et al. (2008) had shown that even though all inflammatory myopathies displayed ubiquitous overexpression of degeneration-associated markers, only in sIBM, expression of APP transcripts were significantly and routinely correlated with inflammation in the muscle [18]. Furthermore, in sIBM, IHC analysis revealed that inflammatory mediators including IL-1 beta co-localized to beta-amyloid depositions within myofibres [18]. In fact, within human myotubes, an upregulation of APP with subsequent intracellular aggregation of beta-amyloid was observed following exposure to interleukin (IL)-1 beta [18], suggesting that production of high amounts of pro-inflammatory mediators in sIBM muscles specifically induces beta-amyloid-associated degeneration. The same correlation was observed in another separate study where it was shown that alphaB-crystallin is associated with overexpression of APP in sIBM muscle and that upregulation of alphaB-crystallin precedes accumulation of beta-amyloid [19]. Our study also supports the decade old hypothesis that in IBM muscle, pro-inflammatory cytokines secreted by immune system cells results in APP production by myofibres, and APP production by myofibres results in a feed-forward feedback loop resulting in inflammatory cytokine secretion by immune cells [20], highlighting that cell stress, inflammation and degeneration all contribute to the pathogenesis sIBM. Cumulatively, the aforementioned observations collaborate our current findings and convincingly shows that β-amyloid protein in sIBM patient muscle sample is not an artifact as occasionally debated [21]. It is plausible that APP induces ERS and UPR, which in turn enhance αB-crystallin expression and subsequent formation of soluble neurotoxic oligomers [22].

During analysis of the KEGG pathway, we found proteins with altered expression levels that are components of 19 pathways. The tight junction pathway, muscle contraction, and Huntington's and Parkinson's disease pathways had the most hits. Aside from these, however, using functional analysis we found that peroxydase1 and αB-crystallin are correlated with the activation of mitogen-activated protein kinase (MAPK), which is primarily involved in regulating cell growth. We surmise that the two proteins regulate the strength of upstream signaling of the MAPK pathway, thus playing an indirect role in regulating cell growth during interactions with molecules such as oxidative stress or extracellular growth factors.

DAVID functional analysis showed that the proteins with different expression patterns in s-IBM participating in regulation of muscle contraction were subunit 1 of troponin T, αB related HSP, myoglobulin L-chain 2, troponin T3, and troponin I. Expression levels of troponin T3 and troponin I were decreased in s-IBM samples, while the other proteins were increased. This result suggests that regulation of muscle contraction is increased during the development of inclusion body myositis.

One limitation of the current study is the small sample size analyzed. This necessitates verification of our findings in a larger cohort of IBM patients, in isolation and in comparison to other myopathy and normal controls.

## Conclusions

In summary, 29 proteins exhibiting differential expression levels in s-IBM were screened in our study. Some of these proteins have not been reported previously. The functions of these proteins include oxidative stress, regulation of apoptosis, signal transduction, and cytoskeleton. Further research into muscle diseases using proteomic methods may provide important clues for better understanding of the pathogenesis of these diseases, and help with more accurate diagnosis.

## Methods

### Patient inclusion criteria and sample collection

All patients provided signed informed consent, and the study was approved by the Institutional Review Board. We used the European Neuromuscular Centre (ENMC) (2011) diagnostic criteria for diagnosis of IBM and subsequent inclusion in the current study. All the included patients met the diagnostic criteria for ‘clinically defined IBM’. The clinical features were duration of weakness > 12 months, creatine kinase (CK) ≤ 15x ULN (upper limit of normal), age at onset > 45 years, finger flexion weakness > shoulder abduction weakness, and knee extension weakness ≥ hip flexor weakness. The histopathological features associated with one or more, but not all of endomysial inflammatory infiltrate, upregulation of MHC class I, rimmed vacuoles, protein accumulation or 15–18 nm filaments. Samples of inclusion body myositis (IBM) were chosen from three IBM patients who first visited the clinic service of our hospital. Biopsy specimens of biceps or quadriceps muscle tissue were selected based on electromyogram results, then washed with saline. Three cases, who first came to our clinic for weak limb and finally pathologically identified as neurogenic muscular atrophy (NMA) by electromyography and normal tissue by muscle biopsy, served as the control group. After blotting of excess saline with filter paper, the tissues were stored at −80°C.

### Whole protein extraction

Preserved muscle preparations were prepared and washed with phosphate buffered saline (PBS) to remove adherent hemoglobin and other contaminating proteins resulting from haematolysis during specimen collection. Subsequently, tissue was homogenized in protease inhibitor-containing lysis buffer (40 mM Tris–HCl, 7 M urea, 2 M sulfourea, 4% CHAPS, 1% DTT, and 1 mM EDTA) at a ratio of 50:1 (buffer: tissue). DNase (10 μg/μl) and RNase (5 μl) were added. Samples were incubated on ice for 20 min, followed by centrifugation at 14000 r/min at 4°C for 20 min. The supernatant which containing the whole protein of muscle was collected and the concentration of total protein was normalized for the subsequent assay.

### Two-dimensional gel electrophoresis

Isoelectric focusing electrophoresis was performed with an Ettan IPGphor IEF System (GE Healthcare, U.S.). Before detection, protein samples were centrifuged for 2 min, then 100 μg of sample was dissolved in 800 μl of rehydration buffer (8 M urea, 0.02% CHAPS, 0.02 M DTT, and 0.05% IPG buffer). For first dimension electrophoresis, 18 cm solid phase pH gradient (pH 3–10) dry adhesive tape was rehydrated for 16 h at 21°C, and 20 μL of sample supernatant was added. Electrophoresis was performed for 15 h at 0-3500 V, followed by electrophoresis for 6.3 h at 3500 V. The focus adhesive tape was balanced twice in SDS balanced solution, and then agitated in a rocking bed for 2x15 min. The adhesive tape was removed for the second phase of vertical SDS-PAGE, with continuous flow at 40 mA for 40 min, and 60 mA 5 h until the leading edge of bromochlorophenol blue reached the bottom of glass plate. Following completion of electrophoresis, coomassie brilliant blue staining was carried out. The 2D gel image was analyzed by ImageMaster 2D Platinum software; analysis and adjustments included intensity correction, spot detection, and background reduction.

### Enzymolysis and mass-spectrum identification and alignment

Differential protein particles were cut from the gel for trypsin digestion. The extracted peptides were dissolved in 0.1% TFA/α-cyano-4-hydroxy-cinnamic acid groundmass, and samples were applied to the target plate. ABI Voyager DE Pro was used for MALDI-TOF/MS analysis. Database queries were carried out via SwissProt, UniProt, and NCBInr on http://prospector.ucsf.edu/prospector/cgi-bin/msform.cgi?form=msfitstandard and http://www.matrixscience.com/.

### Western blotting and RT-PCR

Western blotting and RT-PCR were performed following standard procedures. Anti-APP monoclonal antibody (1:500; Santa Cruz Biotechnology), anti-αB-crystallin polyclonal antibody (1:1,000; Abcam) were used as an internal loading control.

### Statistical analysis

Statistical analysis was performed using SPSS 13.0. Data was represented as mean ± standard deviation (SD). Unpaired *t* test was used to compare groups, P < 0.05 was considered statistically significant.
